# Belief Bias in Individual and Collective Reasoning

**DOI:** 10.5964/ejop.12041

**Published:** 2024-11-29

**Authors:** Alba Massolo, Mariel Traversi, Matías Alfonso

**Affiliations:** 1 Facultad de Filosofía y Humanidades, Universidad Nacional de Córdoba, Córdoba, Argentina; 2 Facultad de Ciencias de la Salud, Universidad Católica de Córdoba, Córdoba, Argentina; Dublin City University, Dublin, Ireland

**Keywords:** deductive reasoning, belief bias, collective reasoning, cognitive biases, group problem-solving

## Abstract

In this paper, we investigate whether collaborative group performance is better than individual performance in solving a syllogism evaluation task. We hypothesise that collaborative group settings will outperform individual settings and that the belief bias effect will be mitigated in a group setting. Two empirical studies were conducted with Argentinian undergraduate students. Study 1 (*N* = 239) used a between-subjects design with two conditions: individual resolution and interactive group resolution. Overall, the group condition performed better than the individual condition, but there were no significant differences in evaluating invalid syllogisms. Study 2 (*N* = 115) used a within-subjects design with three conditions: individual resolution, interactive group resolution, and individual after-interactive group resolution. Overall, the group condition performed better than the individual condition, and the individual after-interactive group condition showed an increase in accurate answers compared to individual resolution. However, as observed in Study 1, the collaborative group setting did not improve the evaluation of invalid syllogisms. We propose an explanation for the group resolution of invalid believable syllogisms within the framework of the selective processing model of the belief bias. This research provides new data on the effects of collaborative settings in deductive reasoning beyond the Western Educated Industrialised Rich Democratic (WEIRD) cultures.

Since the Modern Age, the prevailing view of human reasoning has maintained that reasoning is a solitary process, i.e., a private mental act consisting in accepting or rejecting a conclusion upon evaluating reasons. According to this individualist tradition, private reasoning is highly superior to other forms of reasoning such as collective thinking or group argumentation. Notable figures such as Descartes and Kant strongly advocated in favour of this individualist approach to reasoning (Dutilh-Novaes, 2021). In addition, this approach has had a great deal of influence on the psychology of reasoning, since the vast majority of empirical studies in this field were designed to analyse individual or solitary solutions to reasoning problems (Mercier, 2018).

Nevertheless, in the last years, the philosophy and psychology of reasoning have taken a social turn: in both disciplines, a characterisation of reasoning as a social activity is gaining ground (Kalis, 2022). This alternative approach claims that reasoning is a social activity that requires and reconstructs a shared world (Laden, 2012). Specifically, the shared intentionality hypothesis (Tomasello, 2014) and the interactive account of reasoning (Mercier & Sperber, 2017) have emphasised that the aims of reasoning are social in nature. According to this social approach, the internal process of solitary reasoning that people carry out daily can be seen as an internalisation of this social process. Thus, reasoning can be understood as an originally external practice that subsequently became an internal one (Dutilh-Novaes, 2021).

Decades of research in the psychology of reasoning have amply documented that human beings are defective reasoners when solving deductive problems (Elqayam & Evans, 2011). It is worth noting that nearly all of these studies showing deficient deductive reasoning skills were designed to be worked out individually. However, deductive performance in group-solving tasks improves significantly (Moshman, 2021). Since the 1980s, an increasing number of studies have been conducted to analyse the characteristics of group reasoning (Laughlin, 2011). A group problem-solving and decision-making continuum with intellective and judgmental tasks as two extreme points has been proposed (Laughlin & Adamopoulos, 1980). While intellective tasks have a demonstrably correct answer within a mathematical, logical, scientific, or verbal conceptual system, judgmental tasks are evaluative, behavioural, or aesthetic judgments that do not have a generally accepted demonstrably adequate solution (Laughlin, 2011). In this continuum, deductive tasks are paradigmatic examples of intellective tasks since determining whether an argument is deductively valid or invalid depends on a well-defined formal system and has a unique correct answer within that system.

A series of studies about verbal and mathematical intellective tasks conducted to analyse the difference between individual and collective responses have shown that the performance of groups was considerably better than that of individuals (Laughlin & Adamopoulos, 1980; Laughlin & Ellis, 1986). A similar situation can be observed regarding deductive reasoning tasks. For instance, in a problem of disjunctive reasoning resolved first individually and later in groups, the reported results show that groups outperformed individuals (Trouche et al., 2014). Analogous results were obtained in a Japanese sample (Boku et al., 2018). Furthermore, it was shown that the exchange of arguments during the group resolution process was the main factor explaining the superiority of group performance rather than the subjective confidence of each participant (Trouche et al., 2014).

In a similar vein, comparisons of individual and group performance in the Wason selection task, one of the most replicated tests in the study of deductive reasoning, are in line with these previous results (Moshman, 2021). While accurate answers are around 9% in solitary resolutions of this task, correct responses amount to 70% in the interactive group condition (Moshman & Geil, 1998). Although the increase in correct responses was not as dramatic as in this previous study, the strength of group problem-solving was also reported in Maciejovsky and Budescu (2007), with 9% vs. 50% of correct responses in the individual and group conditions, respectively, and in Mercier et al. (2016), with 20% vs. 63% for these two same conditions. Conclusions also emphasised that the superiority of group performance was due to collaborative reasoning rather than imitation or pressure among group members (Moshman & Geil, 1998). Furthermore, it should be mentioned that most people, not only layman participants but also experts in the field of the psychology of reasoning, underestimate the beneficial effects of collaborative group problem-solving in the performance of the Wason selection task (Mercier et al., 2015).

These studies have typically been conducted using samples of participants from Western, Educated, Industrialised, Rich, and Democratic (WEIRD) cultures. However, this restriction in the sample’s participants limits the generalisability of the results to the broader population. Some recent studies have attempted to work with samples not exclusively from the United States or Europe, acknowledging that reasoning performance may vary across different cultures (Boku et al., 2018; Castelain et al., 2016). Following this line, it is crucial to continue expanding this research to encompass data from countries and communities where information on reasoning performance is scarce or absent.

In this line of research, it is implicitly assumed that group members cooperate and work together to achieve a common goal. Nevertheless, there exists empirical evidence suggesting that not only cooperation but also competition among group members improves performance in the resolution of the Wason selection task (Maciejovsky & Budescu, 2007).

Along with the Wason selection task, another deductive task that has also been widely replicated since the 1980s is the syllogism evaluation task (Ball & Thompson, 2018; Evans et al., 1983; Thompson & Markovits, 2021). This deductive task was designed to analyse the effects of factual content about the world on deductive reasoning. Specifically, the task consists of a series of arguments, generally in the form of categoric syllogisms, some valid and some invalid, some with believable conclusions, and some with unbelievable conclusions. And participants are asked to evaluate whether each argument is valid or invalid (Caravona et al., 2019; Erceg et al., 2019; Ghasemi et al., 2022). Results have shown that there exists a strong effect of content, in the sense that syllogisms with believable conclusions tend to be evaluated as valid, while syllogisms with unbelievable conclusions tend to be evaluated as invalid, regardless of their real logical validity (Evans et al., 1983). This phenomenon is currently known as belief bias and highlights the strong effect of previous beliefs on argument evaluation. Besides, this pervasive cognitive bias heavily influences everyday human reasoning (Ball & Thompson, 2018). Nevertheless, as far as we know, the effects of belief bias on reasoning have been tested exclusively in syllogism evaluation tasks solved individually. There does not seem to be previous research on belief bias in collective group reasoning (Dutilh-Novaes, 2021).

This paper aims to analyse whether the individual and the collaborative group settings have similar effects on the resolution of the syllogism evaluation task with factual content about the world. Based on the previous research on group problem-solving mentioned above, our hypothesis claims that collaborative group performance will be better than individual performance on the syllogism evaluation task. Hence, we also predict that the belief bias effect will be mitigated in the collaborative group setting. To achieve this, we designed and conducted two empirical studies. Study 1 employs a between-subjects design with two conditions: individual resolution and interactive group resolution of the syllogism evaluation task. The goal is to analyse the deductive performance of both conditions. Study 2 uses a within-subjects design with three conditions: individual resolution, interactive group resolution, and individual after-interactive group resolution of the syllogism evaluation task. The goal of this second study is to analyse the deductive performance of the individual and group conditions using a different methodological design and, at the same time, analyse the effects of group discussion in the after-interactive group resolution condition. Moreover, as our sample is composed of Argentinian participants, a further contribution of this research is to provide data from a country not belonging to the WEIRD culture. These results may help to obtain more representative samples to generalise findings on human reasoning.

## Study 1

### Method

#### Participants and Design

The sample size was determined before the start of data collection using the G*Power statistical program; the result was 88 participants per group, i.e., a total of 176 participants, *f* = .5, α = .05. The total non-randomised sample consisted of 239 Argentinian undergraduate students aged between 18 and 58, *M* = 23.32, *SD* = 6.18. Most of them (78.2%) were females, 20.9% were male and two participants indicated belonging to another gender (0.9%). They were enrolled in diverse bachelor's degree programs (Psychology 66.9%, Biology 19.7%, and Criminology 13.4%). All of them were volunteers who did not receive any payment for participating in the study. Participants were evaluated in their classrooms after signing an informed consent, in which the goals of the research were explained, and confidential treatment of the data provided was guaranteed. A between-subject design was used to perform the experiment. Participants were randomly assigned to one of the two experimental conditions: individual resolution and interactive group resolution. Thus, 119 participants performed the task individually and 120 in groups.

#### Materials

An argument evaluation task was administered in Spanish, the native language of the sample. An English version of the task is available in Massolo et al. (2024). The task consisted of a total of eight categorical syllogisms that had factual content about the world. Of these arguments, four were valid and four were invalid, including four with believable conclusions and four with unbelievable conclusions. In this way, the test was composed of two valid believable (VB), two valid unbelievable (VU), two invalid believable (IB), and two invalid unbelievable (IU) syllogisms. An example of each syllogism type is shown in Table 1. Participants were asked to evaluate whether each syllogism was a logically valid or invalid argument.

**Table 1:** Examples of Types of Syllogisms Used in the Syllogism Evaluation Task

Believability	Valid Form	Invalid Form
Believable	No dog is a feline. Some mammals are felines. Therefore, some mammals are not dogs.	No writer is an actress. Some famous women are actresses. Therefore, some writers are not famous women.
Unbelievable	Some wolves have elongated forelimbs. All animals that have elongated forelimbs fly. Therefore, some wolves fly.	No lion is a domestic animal. Some felines are domestic animals. Therefore, some lions are not felines.

Before conducting the study, the mean scores of the believability of the syllogism conclusions were calculated based on the results of a survey answered by a group of 47 participants who did not participate in the present study. Each conclusion was rated on a Likert scale from 1 (‘I am totally sure that it is false’) to 7 (‘I am totally sure that it is true’). The score difference was noticeable: while believable conclusions scored a mean of 5.97, *SD* = 1.13, the mean score for the unbelievable conclusions was 1.7, *SD* = 0.94.

#### Procedure

Participants were randomly assigned to one of the two experimental conditions. Each participant was given a paper copy with an informed consent sheet, a brief explanation of the general objectives of the research, and the argument evaluation task. Subsequently, some demographic questions were asked. In the first part, a brief explanation of the syllogism evaluation task was presented along with examples of both valid and invalid arguments. Also, the two possible answer options for each of the test questions were made explicit as follows: ‘Yes, the conclusion follows logically’ or ‘No, the conclusion does not follow logically’. The arguments were displayed one by one on the page, in random order.

In the individual resolution condition (IR), each participant was asked to read the instructions and evaluate the task, indicating the answer they considered more appropriate. Instead, in the interactive group resolution condition (GR), the participants read the instructions individually. Afterwards, they were randomly assigned to groups consisting of three to five members. Each group was instructed to discuss each syllogism and reach a consensus before giving their answer. Group size was determined based on previous studies (Boku et al., 2018).

### Results and Discussion

**Figure 1:**
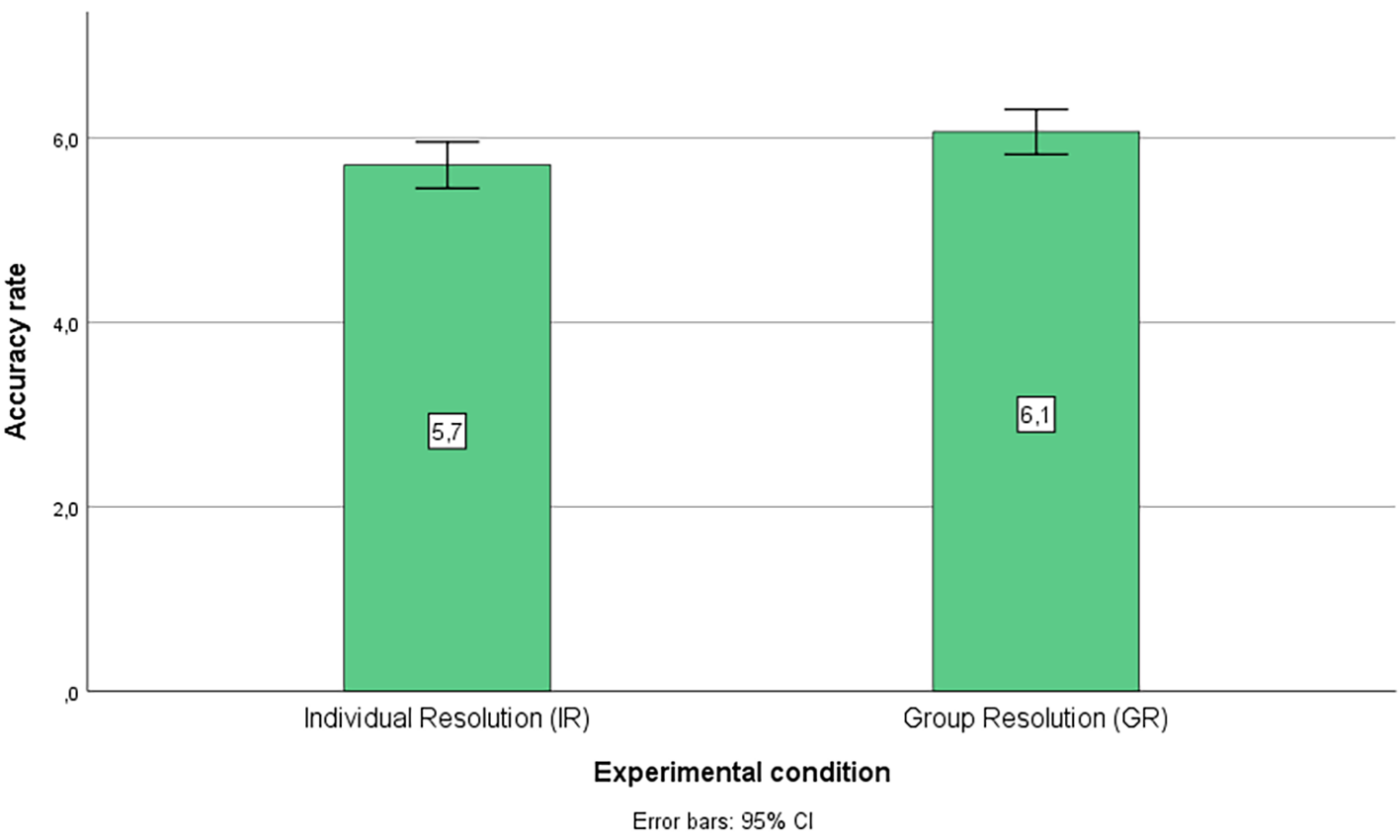
Percentages of Global Accuracy for Each Condition

**Figure 2:**
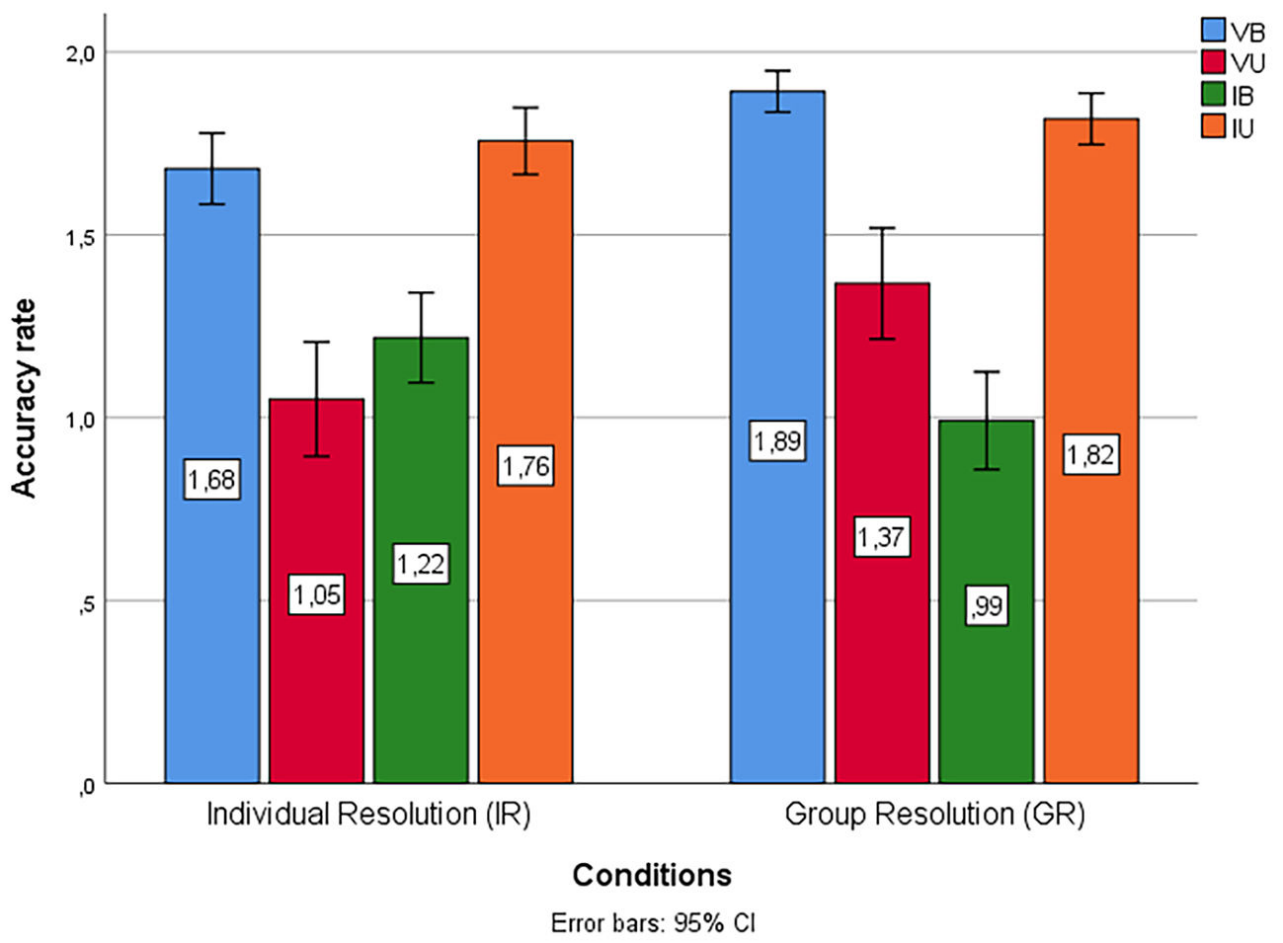
Percentages of Accuracy Scores for Each Type of Syllogism Across the Two Conditions

The gathered data were evaluated using the SPSS v.25.0 software. All processed data is available in Massolo et al. (2024). To evaluate the validity of the hypotheses mentioned above, we analysed mean percentages of the accuracy scores of each type of syllogism for both conditions. In the IR condition, the accuracy rates were 68.28 for valid syllogisms; 74.37 for invalid; 72.48 for believable, and 70.17 for unbelievable. For the GR condition, the accuracy rates were 81.46 for valid syllogisms; 70.21 for invalid; 72.08 for believable, and 79.58 for unbelievable. These data can be seen in Figure 1 and Figure 2. The data are available as supplementary materials.

To statistically analyse the effects of logic and belief, as well as the interaction between them, we calculated the logic, belief, and interaction indexes taken from Evans and Curtis-Holmes (2005), as follows:

Logic index: (VB + VU – IB – IU)
Belief index: (VB + IB – VU – IU)
Interaction index: (VU + IB – VB – IU)

*T*-tests were conducted to determine whether each of the calculated indexes was significantly higher than zero. In the IR group, all three indexes are positive and significantly above zero. In the GR condition, all three indexes were also positive and those for logic and belief were significantly above zero, although the interaction effect was non-significant. We also conducted two-sample *t*-tests to compare logic and belief indexes between the two groups. The logic index scores were significantly lower in the IR group, *t*(237) = -2.04, *p* = .04, *d* = .361, while there was no significant difference in the belief index.

Thereafter, we calculated *t*-tests to compare the overall performance in both conditions concerning accuracy (total number of correct answers), where GR answered more syllogisms correctly than the IR condition, *M*(GR) = 6.07, *M*(IR) = 5.70, *t*(237) = -2.04, *p* = .04, *d* = .36. Then, we computed *t*-tests to compare the performance considering each type of syllogism and found significant differences in the case of VB syllogisms, where GR answered more syllogisms correctly than the IR group, *M*(GR) = 1.89, *M*(IR) = 1.68, *t*(237) = -3.72, *p* < .001, *d* = .211; in the case of VU syllogisms, where GR answered more syllogisms correctly than the IR condition, *M*(GR) = 1.37, *M*(IR) = 1.05, *t*(237) = -2.87, *p* = .004, *d* = .316; and in the case of invalid believable (IB) syllogisms, but this time, IR answered more syllogisms correctly than the GR condition, *M*(IR) = 1.22, *M*(GR) = 0.99, *t*(237) = 2.47, *p* = .014, *d* = .227. Regarding the IU syllogisms, there were no significant differences between the conditions, *t*(237) = -1.03, *p* = .30.

As can be seen, the results partially confirm the prediction that the belief bias effect is mitigated in the collaborative group setting. The group resolution condition obtained more correct answers in general, but considering the four types of syllogisms, this is true only for the case of VB and VU syllogisms.

As significant differences have been observed between the conditions analysed, in the next study, we carry out a similar analysis with a within-subjects design, where the instrument is applied individually before and after an instance of group interactive resolution to observe the effects of group discussion in relation to the accuracy in the response. This design allows further analysis of the variations between the individual and the group interactive resolutions, as well as whether the group reasoning stage affects the individual after-interactive group resolution of the reasoning task.

## Study 2

### Method

#### Participants and Design

The goal of this study is to analyse the deductive performance of individuals and groups using a within-subject design with three conditions: (1) individual resolution, (2) interactive group resolution, and (3) individual after-interactive group resolution of the syllogism evaluation task. Besides, this design makes it possible to examine the effects of group discussion in the after-interactive group resolution condition. G*Power software estimated a sample size of 42 participants, *f* = 0.25, α = .05. A non-randomized sample of 115 Argentinian undergraduate students aged between 18 and 54, *M* = 19.70, *SD* = 3.69, was analysed. None of the participants included in this study were part of the sample from Study 1. Most of them (77.4%) were females, 20.9% were male and two participants indicated belonging to another gender (1.7%). They attended undergraduate programs in psychology and were volunteers who did not receive any payment for participating in the present study. They were evaluated in their classrooms after accepting informed consent, in which the goals of the research were explained, and confidential treatment of the data provided was guaranteed.

#### Materials

The instrument was the same one used in Study 1.

#### Procedure

Prior to beginning the task, participants read a set of general instructions for solving the problems. The study consisted of three stages. In the first stage, participants solved the argument evaluation task individually indicating the answer they considered more appropriate: ‘Yes, the conclusion follows logically’ or ‘No, the conclusion does not follow logically’. This stage corresponds with Condition 1. In the second stage, Condition 2, participants were randomly assigned to groups of 3 to 5 participants and moved to another classroom to interact in the group setting and solve the task. Participants were then instructed to reach a single decision for each syllogism response after a group discussion instance Finally, in the third stage, Condition 3, participants were asked to solve the argument evaluation task individually again.

### Results and Discussion

The gathered data were evaluated using the SPSS v.25.0 software. We calculated the indexes for logic, belief, and interaction. *T*-tests were carried out to determine whether each of the indexes was significantly higher than zero, and the results were positive for all indexes. We also conducted two repeated measures analyses of variance to compare the logic and belief indexes across the three conditions. The three conditions (1, 2, and 3, respectively) were entered as within-group factors levels. There was a main effect, *F*(1,495) = 4.60, *p* = .02, η*^2^* = .167, pairwise comparisons indicate that the logic index scores were significantly higher in Condition 2, *M* = 2.25, *SD* = 1.15, than in Condition 1, *M* = 1.42, *SD* = 1.25, *p* = .05, and Condition 3, *M* = 1.83, *SD* = 1.27, *p* = .04, while there was no significant difference in the belief index scores. The data is available in Massolo et al. (2024).

**Figure 3:**
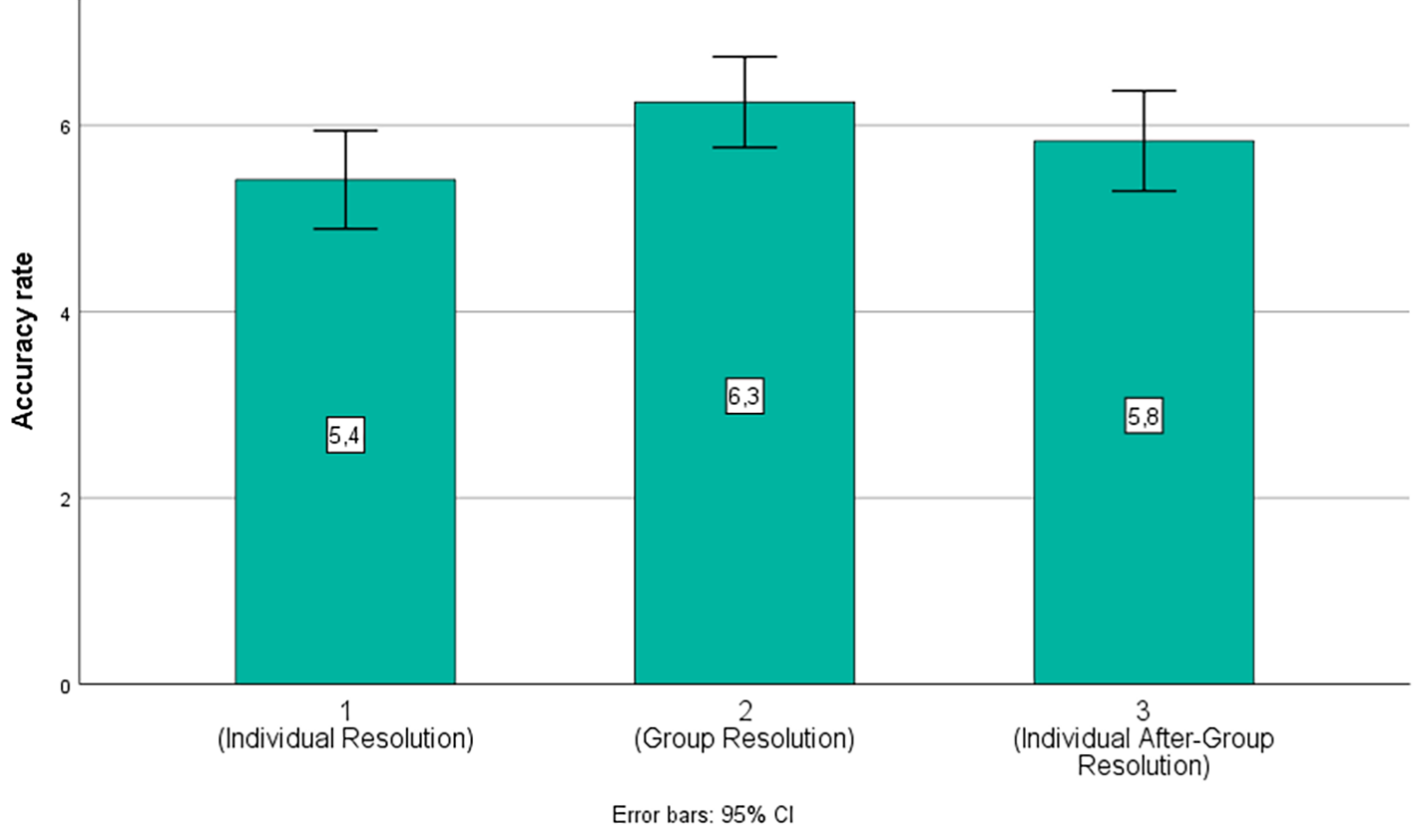
Percentages of Global Accuracy for Each Condition

**Figure 4:**
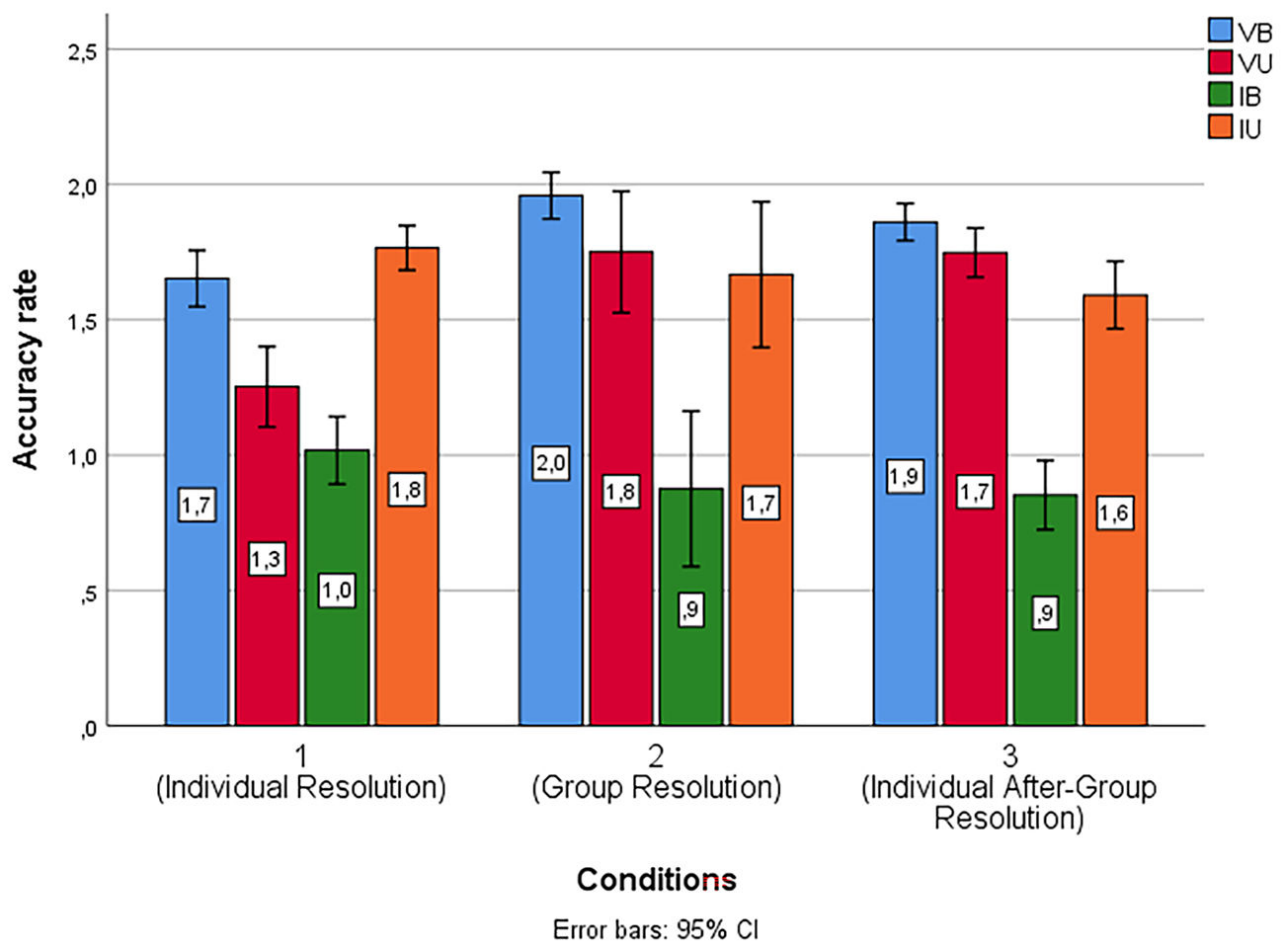
Percentages of Accuracy Scores for Each Type of Syllogism Across the Three Conditions

The percentages of global accuracy scores, and for each type of syllogism across the three conditions, are displayed in Figures 3 and Figure 4, respectively. As can be seen here, the highest number of correct responses for valid syllogisms was observed in Condition 2.

Next, we conducted a within-subject repeated measures ANOVA to compare accuracy scores across different conditions. Overall, we found a main effect, *F*(1,495) = 4.60, *p* = .02, η*^2^* = .167, with a significantly higher mean accuracy score observed in Condition 2, *M* = 6.25, *SD* = 0.23, than in Condition 1, *M* = .41, *SD* = 0.25, *p* = .05. No statistically significant differences were observed for Condition 3, *M* = 5.83, *SD* = 0.26.

Moreover, we computed within-subject repeated measures ANOVAs to compare accuracy scores for each type of syllogism separately, and significant differences were found only in the case of valid syllogisms. Specifically, more VB syllogisms were correctly responded to in Condition 2, *F*(1,725) = 7.00, *p* = .004, η*^2^* = .233, *M* = 1.96, *SD* = 0.04, than in Condition 1, *M* = 1.36, *SD* = 0.15, *p* = .008; and more VU syllogisms were correctly responded in condition 2, *F*(1,251) = 7.14, *p* = .008, η*^2^* = .237, *M* = 1.75, *SD* = 0.10, than in either Condition 1, *M* = 1.33, *SD* = 0.17, or Condition 3, *M* = 1.70, *SD* = 0.11, *p* = .04. However, in Condition 3 there were significantly more correct responses than in Condition 1 for VU syllogisms. Regarding performance on IB and IU syllogisms, no differences were found between the mean accuracy scores. *F*(1.071) = .324, *p* = .590; *F*(1,793) = 1.15, *p* = .369, respectively. It is worth noting that, although the difference was not significant, Condition 1, *M* = 0.96, *SD* = 0.75, had a higher number of correct responses for IB syllogisms compared to Condition 2, *M* = 0.88, *SD* = 0.68, as occurred in Study 1.

The current findings are consistent with those of Study 1, as they reaffirm the expectation that belief bias is diminished during group exchange. Overall, the results suggest that group resolution yielded superior performance compared to individual resolution; however, no significant differences were observed for invalid syllogisms. Notably, a significant enhancement in individual performance was noted after group exchange, but only for VU syllogisms. Consequently, it can be concluded that the hypotheses were partially supported.

## General Discussion

The main objective of the present research was to determine whether collaborative group problem-solving would result in better performance in evaluating syllogisms with factual content compared to individual problem-solving. Additionally, this research aimed to investigate whether the effect of belief bias could be reduced in the collaborative reasoning setting. Overall, the results obtained in the two studies show that the collaborative group resolution of the syllogism evaluation task outperformed the individual resolution. Thus, as a corollary, belief bias is slightly mitigated in collaborative group problem-solving. In this way, the results obtained partially confirm our initial hypothesis, as they exhibit distinct characteristics that warrant further examination.

Firstly, the collaborative resolution of the syllogism evaluation task in Study 1 did not show as dramatic an improvement as the group resolution of the Wason selection task reported in previous studies such as Moshman and Geil (1998). Similarly, in Study 2, while the interactive group condition performed better than the individual resolution condition, the improvement in deductive performance was modest. All in all, these results suggest that the improvement in group resolution observed in the syllogism evaluation task is not as strong as in other deductive and intellective tasks.

Secondly, a separate analysis of the resolution of the four types of syllogisms reveals that the group resolution demonstrates superior performance only in the case of valid syllogisms. This effect is particularly striking for IB syllogisms, where results show not only that group resolution fails to yield any significant improvement over individual resolution, but also that individual resolution outperforms group resolution, as evidenced by higher accuracy mean rates. These findings were obtained both in Study 1 and Study 2. In line with these results, it was shown that belief bias occurs more often in the evaluation of invalid syllogisms than in valid syllogisms (Evans et al., 1983). And what is more, according to the selective processing model, IB syllogisms exhibit the highest rate of incorrect responses (Stupple et al., 2011). The selective processing model is a dual-process model that posits the involvement of two different types of processes in reasoning: a default response driven by previous knowledge and beliefs, and a response based on motivated reasoning driven by the believability or unbelievability of the syllogism conclusions (Ball & Thompson, 2018). According to this model, the default response is to consider valid syllogisms with believable conclusions and to consider invalid syllogisms with unbelievable conclusions. This explains the belief index (Evans et al., 2001). However, this default response can be overridden by the second type of processing. Hence, if a motivated reasoning occurs, and there is a believable conclusion, reasoners will try to construct a single model that supports the argument. Whereas if there is an unbelievable conclusion, reasoners will try to construct a single model that refutes the argument (Ball & Thompson, 2018). This explains the interaction between logic and belief. Thus, in the case of valid arguments, unbelievable conclusions tend to motivate the search for a countermodel, i.e., a model where the premises are true, but the conclusion is false. However, as the syllogism is valid, this countermodel cannot be found. This limits the influence of belief bias (Stupple et al., 2011). In the case of invalid arguments, believable conclusions tend to motivate the search for a model showing the consistency of the premises and the conclusion. Nevertheless, for invalid arguments, models can exist where both premises and conclusion are true. Hence, if reasoners construct this supportive model, they erroneously answer that the argument is valid. This explains the high levels of inaccurate responses for IB syllogisms.

At present, the selective processing model is considered one of the most robust theoretical models for explaining belief bias effects in reasoning, as noted by Ball and Thompson (2018). Although this model was not originally designed to explain deductive reasoning in a collaborative setting, it is possible to hypothesise that if all members of a reasoning group searched for a model that supported the conclusion in the case of IB syllogisms, they would be more likely to find such a model. This could explain why the interactive group condition did not show improvement in resolving IB syllogisms.

Thirdly, in both studies, the logical index is higher in the group condition. This shows that collaborative group settings give rise to the elaboration of more logically accurate responses compared to solitary reasoning. These findings are in line with theoretical developments that vindicate a dialogical conception of deduction since they reveal that humans become better reasoners when making deductive inferences in collaborative settings (Dutilh-Novaes, 2021). Besides, in the case of Study 2, the individual after-group resolution condition, i.e., Condition (3), shows a better performance than the individual resolution condition, i.e., Condition 1, for some syllogisms. This suggests that reasoners manage to maintain the improvements achieved during the group resolution stage for the evaluation of VU syllogisms.

This research offers novel insights by analysing the collaborative resolution of a syllogism evaluation task in a sample of Argentinian participants. This is particularly noteworthy since deductive reasoning tasks have traditionally been studied in Western, developed countries, often belonging to the WEIRD culture. Results have shown that individual performance in the syllogism evaluation task is broadly similar in the Argentinian sample compared to the WEIRD samples analysed in previous studies. Besides, improvements in group reasoning have also been observed in the Argentinian sample. However, due to the absence of prior data on group resolutions of the syllogism evaluation task, direct comparisons with our findings are not feasible. All in all, incorporating empirical findings from samples comprising participants across diverse countries and cultures will advance our understanding of human deductive reasoning.

Regarding the limitations of our studies, firstly, the samples were drawn exclusively from undergraduate students, which may have curtailed the diversity of our samples. For future research, it is desirable to include people of different ages, educational levels, and cultural backgrounds. Secondly, as noted by one of the anonymous reviewers, the variability in group sizes, ranging from three to five participants, in both studies might have affected the results. This is because a majority rule may have been used to solve the task in groups with an odd number of participants. However, a rule like this could not have been applied in groups with an even number of participants. Attending this observation, further research could be conducted to analyse the impact of group size on collaborative reasoning. Even so, the data presented in this study make a valuable contribution to our understanding of the effects of collaborative settings on deductive reasoning.

## Supplementary Materials

For this article, the following Supplementary Materials are available:

- Codebook for Studies 1 and 2. (Massolo et al., 2024)
- Syllogism evaluation task for Studies 1 and 2. (Massolo et al., 2024)
- Syntax code for Studies 1 and 2. (Massolo et al., 2024)

## Data Availability

For this article, data, codebook and materials are available at Massolo et al. (2024).
